# Physical Activity Behaviour and Comparison of GPAQ and Travel Diary Transport-Related Physical Activity in Accra, Ghana

**DOI:** 10.3390/ijerph19127346

**Published:** 2022-06-15

**Authors:** Lambed Tatah, Matthew Pearce, Rahul Goel, Soren Brage, James Woodcock, Fidelia A. A. Dake

**Affiliations:** 1 MRC Epidemiology Unit, University of Cambridge, Cambridge CB2 0SL, UK; matthew.pearce@mrc-epid.cam.ac.uk (M.P.); soren.brage@mrc-epid.cam.ac.uk (S.B.); jw745@medschl.cam.ac.uk (J.W.); 2 Transportation Research and Injury Prevention Centre, Indian Institute of Technology Delhi, Hauz Khas, New Delhi 110 016, India; rahulatiitd@gmail.com; 3 Regional Institute for Population Studies, The University of Ghana, Legon, Accra P.O. Box LG 96, Ghana; fadake@st.ug.edu.gh

**Keywords:** transport physical activity, active travel, travel diary, GPAQ, Ghana

## Abstract

There is a lack of data on physical activity (PA), active travel, and the comparison of measurement instruments in low-resource settings. The objective of this paper is to describe PA behaviour and the agreement of walking estimates from the Global Physical Activity Questionnaire (GPAQ) and the travel diary in a low-resource setting. We used a cross-sectional survey design to capture data from the residents of Accra (Ghana) between May 2020 and March 2021. Of the 863 participants aged 15+ years, 65% were females, and 86% reported PA. The median weekly PA was 18 (interquartile range: 5–75) metabolic equivalent of task hours, with 50% of females and 37% of males achieving low PA levels. In the GPAQ, 80% of participants reported weekly walking; the mean number of days walked was 3.8 (standard deviation (SD): 2.5); hence, 54% of participants reported walking on any day, and the mean daily walking duration was 51 (SD: 82) minutes. In the diary, 56% of participants reported walking for over 24 h, with a mean walking duration of 31 (SD: 65) minutes. The correlation of walking duration between instruments was weak (rho: 0.31; 95% Confidence Interval: 0.25–0.37); the mean bias was 20 min, with GPAQ estimates being 0.1 to 9 times higher than diary estimates. We concluded that low PA is prevalent in Accra, and while the travel diary and GPAQ estimate similar walking prevalence, their walking duration agreement is poor. We recommend accompanying PA questionnaires with objective measures for calibration.

## 1. Introduction

Regular physical activity (PA) has well-established health benefits [1,2], but the literature describing PA behaviour in low-resource settings is sparse. A 2008 systematic review on the prevalence of PA in two low- and middle-income countries (LMICs) identified no study on PA in Ghana and only four in Nigeria [3]. A recent scoping review in 2022 corroborates this gap and highlights the lack of recent high-quality data in all 17 articles identified on PA in Ghana, with multiple studies recycling the 2007/2008 World Health Organisation Study on global ageing and adult health [4]. This sparse literature on PA behaviour in LMICs hinders efforts to control non-communicable diseases (NCDs)—the leading global cause of disability and mortality—since PA is a major NCD risk factor and LMICs bear the highest burden of NCDs [5].

Transport PA (primarily walking and cycling, including while accessing public transport) is a major contributor to total daily PA [6], especially in LMICs [7,8]. Population levels of transport PA are commonly captured through surveys that either use domain-specific questionnaires such as the Global Physical Activity Questionnaire (GPAQ) [9] or travel diaries [10]. However, it is unclear how PA estimates from questionnaires compare to those in travel diaries, and this can be problematic for research seeking to use PA estimates from different sources—for example, in modelling the health impact of transport or comparing PA across places and time.

The GPAQ estimates PA from work, leisure, and transport domains and is being used in many LMICs [11,12,13]. Travel diaries capture travel behaviour—how people move to places in terms of the modes used, such as walking and cycling, duration of travel, and purpose for doing so—but are less common in LMICs [14,15]. Both instruments attempt to capture daily active travel duration but accomplish this in different ways. The GPAQ records the number of days per week of walking or cycling for ≥10 min continuously and the total time spent walking or cycling on a typical day [16]. On the other hand, travel diaries aim to record every individual segment of walking and cycling for transport that occurs during a reference day; the sum of these is the daily active travel duration.

The use of these different instruments will typically lead to different estimates of transport PA. The GPAQ places the burden of recalling travel information on the responders by asking them to aggregate and average their daily active travel time. The travel surveys rather guide responders through their daily transport events; they vary in how well they capture purely leisure trips since walking and cycling are not always for or a result of travelling. In addition, travel diaries rarely count walking inside building complexes. The GPAQ, therefore, tends to provide coarser aggregated estimates of all PA compared to the higher time-resolution data of some transport PA captured by travel surveys. Both types of instruments suffer from recall and social desirability biases. High-income settings have sought to reduce recall bias by allowing participants to fill their travel diaries as days progress and by introducing better control mechanisms. Control mechanisms that ensure the correct completion of questionnaires have included pre-survey visits to introduce the survey and questionnaires and follow-up phone calls to remind participants and clarify doubts. These approaches are often seen as too costly for most LMICs, so survey data from these settings may contain higher biases, which will substantially affect the comparison of survey instruments.

Although the GPAQ is widely used, its validity for total PA is poor when compared to accelerometers, pedometers, and PA logs [16,17,18]. Bull et al. [9] investigated the reliability and validity of the GPAQ from nine countries and showed a moderate to substantial strength of reliability (0.67 to 0.73), a moderate to strong concurrent validity compared with the International Physical Activity Questionnaire (IPAQ) (0.45 to 0.65), and a poor to fair criterion validity (0.06 to 0.35). Fewer studies have examined the corresponding reliability and validity of transport surveys for PA, even in high-resource settings [10,19]. Adam et al. [9] report fair reliability of transport PA questionnaires for walking (ICC = 0.59) and cycling for transport (ICC = 0.61); they report a strong agreement for vigorous physical activity (r = 0.72, *p* < 0.001) and a fair agreement for moderate physical activity (r = 0.24, *p* = 0.09) when compared with accelerometers. The focus of these studies has been on high-income settings, limiting the generalisation of findings. It is important to understand the level of agreement between estimates of transport PA from different instruments in multiple contexts to aid comparisons between studies or settings and facilitate data harmonisation.

This study investigates PA behaviour and the agreement between transport PA estimates derived from the GPAQ and travel diary among residents of Accra, Ghana. The specific objectives are to (i) describe the distribution of overall and domain-specific PA by gender and age group and (ii) compare the agreement of walking estimates between GPAQ and travel diary. Our main hypothesis is that both instruments measure the same latent PA behaviour and therefore agree highly.

## 2. Materials and Methods

### 2.1. Study Design and Setting

We undertook a cross-sectional survey of residents of Accra, the capital city of Ghana, to describe their PA behaviour and compare walking estimates between the GPAQ and travel diary. Our data collection lasted from May 2020 to March 2021. The study protocol was approved by the Ethics Committees for the Humanities (ECH), University of Ghana (ECH 036/19-20). We reported our study following the STROBE guidelines [20].

Accra usually represents a broader area of 12 districts, but the city of Accra refers to the Accra Metropolitan District. The administrative boundaries and hence the populations of the districts in Accra have continually been redefined due to political restructuring. Accra is one of the fastest urbanising cities in Africa, with an annual population growth rate of around 2% since the mid-2000s [21] and a daily influx of 2.5 million business commuters [22]. The survey was conducted around the COVID-19 pandemic period. Like most cities around the world, Accra implemented stay-at-home orders to control COVID-19. A three-week partial lockdown in March/April 2020 was enforced by security agencies, and a stay-at-home order applied to almost all people in most occupations. Although we did not collect data during the lockdown period, the movement of people was affected even after lifting the lockdown, as restrictions on gatherings continued, and people were only allowed to carry out essential activities.

### 2.2. Participants

The study participants were residents of Accra. We considered a resident to be a member of any household in Accra who had spent the night that preceded the interview day in the household, including visitors. Participant inclusion criteria were (i) age ≥ 15 years and (ii) residence located within Accra. The exclusion criteria were (i) adolescents below 18 years who resided in households headed by minors (below 18 years) since minors could not provide valid parental or guardian written consent and (ii) residence in an institutional or a business building such as a care home or a hotel.

### 2.3. Sampling

We designed the sample to be representative of urban Accra; thus, we drew samples from the various districts in Accra. We used a two-stage stratified sampling design to select participants. In the first stage, we selected enumeration areas based on the 2010 Population and Housing Census frame to form the primary sampling units. We selected 47 enumeration areas spread across the various districts, with the probability of enumeration area selection proportional to population size. In the second stage, we systematically selected 10 to 15 households from each enumeration area to form the secondary sampling units. We surveyed all eligible individuals in the selected households who were available after three consecutive visits to the household.

We determined that the sample size for the comparison of the prevalence of PA between males and females was 210 participants per gender using the following parameters: an average PA prevalence of 75%, a difference in prevalence between gender of 10%, confidence intervals of 95%, a power of 80%, and a cluster design effect of 2. We determined that a minimum sample size of 300 per gender would allow for coarse age group comparison between genders.

### 2.4. Data Collection

**Figure 1:**
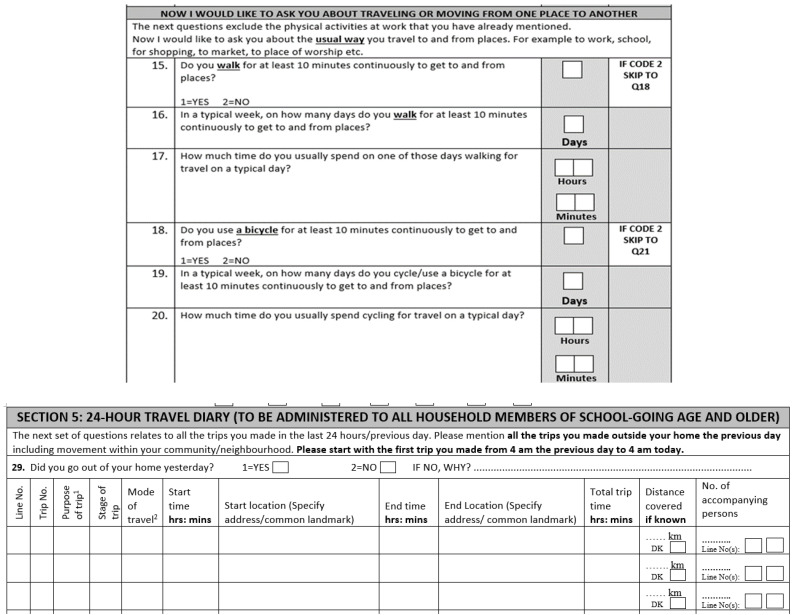
Excerpts of survey questionnaires. The GPAQ (upper panel, which captures data on a typical day and week) shows separate questions for walking and cycling. The travel diary (lower panel, captures data on a specific day, say yesterday) shows included variables.

We collected data using the computer-assisted personal interview (CAPI) approach. We used a household questionnaire and an individual questionnaire to gather information. The household questionnaires captured information on household ownership of any vehicle (yes/no) and a motorised vehicle (yes/no). Individual questionnaires captured information on sex (female, male), age (years in seven categories), education (preschool, primary, middle, secondary, tertiary), occupation (none, student, domestic, paid work), and marital status (never married, formerly married, married). Individual questionnaires included the GPAQ and the travel diary. The GPAQ questions were used mostly in their original form [10], with a slight modification of questions on transportation: we separated walking and cycling, and different questions were asked regarding these two methods of transportation (Figure 1, upper panel). Travel diary questions were adapted from multiple surveys to suit the local context, especially vehicle categories and names (Figure 1, lower panel).

### 2.5. GPAQ Variables

We used data from the GPAQ to estimate minutes per day of moderate-to-vigorous physical activity (MVPA) in total and by the domain (work, leisure, transport). MVPA referred to any activity ≥ 3 MET and included walking, cycling, running, hiking and football games. Sedentary behaviour was collected in hours per day and converted into minutes per day. Examples of sedentary activities included television viewing, playing video games, using a computer, sitting at school or work, and sitting while commuting. The duration of walking or cycling for transport on a typical day was multiplied by the number of days walked or cycled per week and divided by 7 to give the average minutes per day of walking and cycling. Durations of moderate and vigorous physical activity were multiplied by intensity expressed in METs (metabolic equivalent of task) to estimate the total physical activity volume in MET-h/day. Physical activity volume was also categorised as high, medium, and low following the GPAQ guidelines [23].

### 2.6. Travel Survey Variables

We estimated the minutes per day of walking and cycling from the travel diaries by summing all durations of walking and cycling trips performed by individuals during the survey day. The total transport PA was the sum of minutes per day of walking and cycling.

### 2.7. Data Management and Statistical Analysis

We entered data on electronic tablets using the CSPro7 software. The lead field researcher (F.A.A.D) double-checked completed questionnaires at the end of each data collection week. We anonymised the final data and exported them to the R statistical software for regular data quality checks and subsequent data analysis. Only the lead investigator (F.A.A.D) and designated collaborators were able to access the data.

Our exploratory analysis involved using a combination of summary statistics and plots to check for missingness, normality, zero-inflation, and near-zero variance. No data were imputed for missing data. Non-normal dependent variables were log-transformed, while variables with near-zero variance were regrouped when possible.

We used Pearson’s correlation coefficient to evaluate the relationship between active travel duration estimated using the GPAQ and travel diary. Absolute agreement of estimates from the two instruments was assessed by calculating the mean bias and 95% limits of agreement and by plotting the difference between estimates against their mean in Bland–Altman plots [24].

To examine if the time frame difference between methods (7 days vs. 1 day) impacted the correlations, we randomly assigned individuals into groups of seven (n = 40 draws), with each group containing individuals reporting their trip diary on different days of the week, and then compared between-group correlations with individual-level correlations.

## 3. Results

### 3.1. Characteristics of Participants

We surveyed 1159 participants in 602 households in Accra. Eight-hundred and sixty-three (75%) survey participants aged 15+ years were retained for this analysis. The characteristics of these participants are shown in Table 1. The majority (65%) of participants were females. Compared to males, a lower proportion of females had completed at least secondary level education (61% vs. 45%); a higher proportion of females were not working (20% vs. 15%); a lower proportion of females were living in households with any type of vehicle (32% vs. 14%) or with a motorised vehicle (21% vs. 12%).

### 3.2. GPAQ Estimates of Physical Activity

Eighty-six per cent of participants engaged in at least some MVPA, and the median (low-upper quartile) PA was 18 (5–75) MET-h/week. The proportion of participants engaging in some MVPA was similar for females and males, and females’ median PA was half that of males (14 (4–58) vs. 28 (8–120) MET-h/week). Table 2 shows the distribution of MVPA by sex and age groups. Almost half (45%) of all participants were categorised as having low PA volume (4 (0–8) MET-h/week), with a higher proportion of low PA levels in females than in males (50% vs. 37%, *p* < 0.001). Low PA volume was predominant in all female age groups, while high PA volume was predominant in males younger than 55 years.

Participants spent on average (low–upper quartile) 127 (11–150) minutes engaging in MVPA, with females in all age groups reporting fewer minutes than males overall (110; 9–120 vs. 157; 17–208) and in each PA domain. Participants in the 35–44 years age group reported the highest duration of MVPA (Table 3). Work and travel domains contributed the most to the total daily MVPA duration (65; 0–14 and 54; 6–60 min). More time was spent walking than cycling in the travel domain. The daily walking time was 51 (6–60) minutes. Recreational activity contributed the least to total daily MVPA (23%). Females spent more time on sedentary behaviours compared to males (276; 120–360 vs. 261; 150–300).

### 3.3. Travel Diary Estimates of Physical Activity

Just above half (56%) of the participants reported transport PA (walking or cycling). Walking was the main source of transport PA, as only 1% of individuals reported cycling. The average daily transport PA duration was 32 (0–40) minutes, and the daily walking duration was 31 (0–40), equal for both sexes (Table 4).

### 3.4. Comparison of GPAQ and Travel Diary Estimates of Transport Physical Activity

Only 70 (8%) participants reported cycling in the GPAQ and 8 (1%) in the travel diary. Therefore, we focused the rest of our comparison of transport PA only on walking because the number of cyclists was small.

The numbers of participants reporting walking in each instrument are shown in Table 5. In the GPAQ, 694 (80%) participants reported walking over a typical week. The mean (standard deviation) number of days walked per week was 3.8 (2.5); thus, the expected proportion of participants walking on any given day in the GPAQ was 54% (95% CI: 51–58%). In comparison, 482 (56%) participants reported walking over a 24-hour period in the travel diary. The proportion of participants reporting walking in the travel diary was therefore similar to the proportion of participants expected to report walking on any given day in the GPAQ. Of the participants reporting walking on any day in the GPAQ, almost two-thirds (63%) reported walking in the diary. The proportion was only slightly higher (70%) among those who reported walking for five or more days. In both instruments, we found no association between the reporting of walking and age, occupation, education, and access to a vehicle in the household.

Table 6 shows the comparison of daily walking duration from the GPAQ and travel diary. The overall mean bias in daily walking duration for GPAQ minus travel diary was 20 (95% agreement limit: –151 to 192) minutes; the bias increased with the number of days walked in the GPAQ and almost tripled in participants who reported walking for all seven days in the GPAQ (57; –179 to 294 min). The bias was stronger in males (31; –136 to 197 min) compared to females (15; –158 to 188 min) and in those with lower than secondary education (25; –163 to 213 min) compared to those who had at least a secondary education (16; –139 to 172).

**Figure 2:**
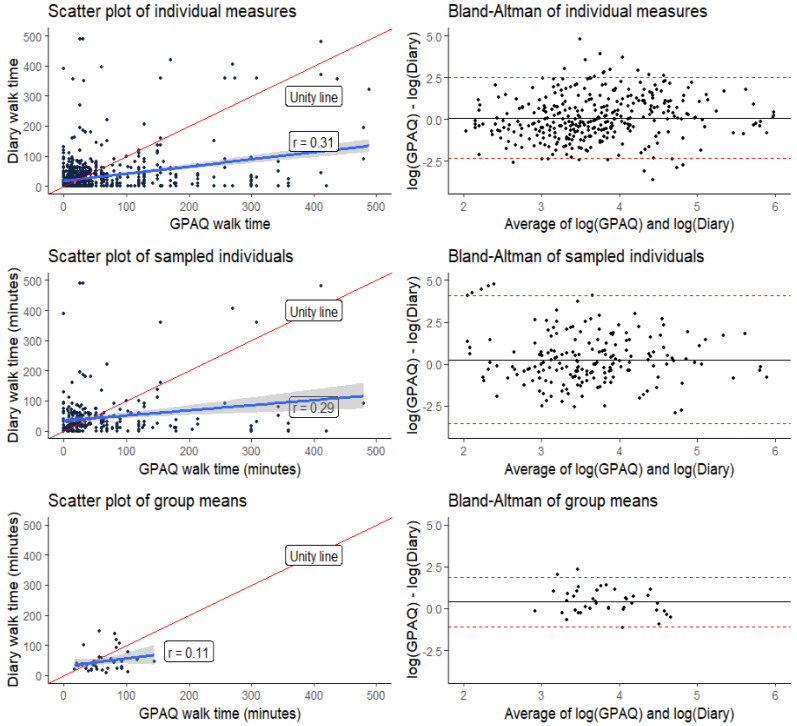
Comparison of walking duration in the GPAQ and travel diary. The left panels show the degree of deviation of the line of best fit from the unity line in the original data (**top**), in the sampled individuals (**middle**), and the group means of groups of seven random individuals with different travel diary days (**bottom**). The right panels show bias and 95% limits of agreement on log scales for original data (**top**), sampled individuals (**middle**), and group mean of sampled individuals (**bottom**).

The Bland–Altman plots (right panel of Figure 2) show substantial disagreement between GPAQ and trip diary daily walking durations. The plots are on a log scale since the original scale showed significant heteroscedasticity. When transformed from the log back to the original scale, the limits of agreement for individual GPAQ and trip diary daily walking durations were 0.1 and 9.0, implying that GPAQ estimates could be between 0.1 to 9 times travel diary estimates. When daily walking duration is capped at three hours, GPAQ estimates could be as high as six times the trip diary estimates.

The overall correlation between the GPAQ and travel diary walking duration was weak (rho = 0.31; 95% confidence interval: 0.25 to 0.37) and decreased as the number of days walked in the GPAQ increased (Table 6). Compared to males, the correlation was weaker in females (42; 32 to 50 vs. 28; 20 to 35).

When we randomly sampled individuals in groups of seven, such that each group contained individuals who were interviewed for their trip diaries on different days of the week, we saw no significant between-group correlation (rho = 0.11, 95% CI: −0.22 to 0.40) compared to a significant within-group positive correlation (rho = 0.29, 95% CI: 0.16 to 0.38). The left panel of Figure 2 shows the deviation of lines of best fit from the line of unity, with a nearly random distribution of group means.

## 4. Discussion

### 4.1. Main Findings

To our knowledge, this is the first study evaluating the agreement of a GPAQ and a travel diary in a low-resource setting. We analysed the PA of 863 participants aged 15+ years; 65% of them were females, and 86% engaged in some PA. The median PA was 18 (IQR: 5–75) metabolic equivalent of task hours per week, with 50% of females and 37% of males having low PA levels. A total of 80% and 56% of participants reported walking in the GPAQ and travel diary surveys, respectively. The proportion of participants expected to report walking on any given day in the GPAQ was 54% (95% CI: 51–58), similar to that in the travel diary. The mean (standard deviation) daily walking time was 31 (65) min in the diary and 51 (82) min in the GPAQ. The mean bias error in daily walking time (GPAQ minus diary) was 20 min (95% limits of agreement: –151 to 192). The bias was stronger in males and participants with lower than secondary education. The correlation of walking time between the instruments was low (r = 0.31; 95% CI: 0.25–0.37), and there was substantial disagreement between the instruments, with daily walking time estimates in the GPAQ corresponding to between 0.1 and 9 times the estimates from the travel diary.

### 4.2. Interpretation of Findings

We observed similarities in the proportions of participants reporting daily walking and in the average daily walking time between the travel diary and the GPAQ when we scaled down the GPAQ to expected daily walking levels. This is an important finding from the point of reporting and monitoring population levels of transport-related PA, especially in the context of limited data, where estimates from one survey would be sufficient. For example, estimates of active travel from a travel diary could inform population levels of transport PA, an important contributor to total PA in LMICs [7], and scaled PA estimates from a GPAQ can inform active travel. Although population PA estimates from either instrument may deviate from objective measurements, validity studies argue a place for these instruments in population PA evaluation [9,10,25].

On the other hand, we observed a poor correlation and a substantial disagreement in walking time estimates between the two instruments. One could expect a poor correlation and disagreement between instruments in a low walking context. Marked differences will result in such contexts because the daily walking time in the GPAQ is an average across one week, while the travel diary reports for a single day. As a result, there will be many people with daily walking times in the GPAQ but with zero walking time in the travel diary. However, in a context with high walking rates, such as Accra, a higher correlation and agreement are to be expected.

The disagreement between instruments was different across population subgroups, with the absolute mean difference in daily walking time (GPAQ minus travel diary) being higher in males and those with lesser education. The reason for population subgroup differences in PA reporting is unclear, but there is some evidence about gendered differences in memory, with females tending to exhibit better recall of activities than males [26,27]. A low level of education has been associated with lower validity of reported physical activity [28].

Studies report conflicting results on the validity and reliability of PA questionnaires with regard to their timeframes. PA questionnaires that target a usual week within a quarter-year or year seem to have better test–retest reliability compared to those targeting an actual week, as the usual week questionnaires control for week-to-week variation in PA patterns [29]. Minimal seasonal influence has been reported on PA questionnaires that target a one-year time frame [30]. A systematic review by Doma et al. [31] further shows that the test–retest reliability of actual and usual week PA questionnaires are rather comparable, partly because of the different reliability cut-offs used in studies. Similar to the usual vs. actual week comparison, weeklong PA questionnaires can seem to average day-to-day variations in PA patterns, but we did not see any significant between-day correlation to suggest that the survey day affected the correlation between average daily walking time in the GPAQ and travel diary. This allows us to conclude that the degree of the agreement we see between these two assessment methods is not primarily influenced by large day-to-day fluctuations.

Overall, a plausible explanation of the bias we see is that people inconsistently overestimate walking when asked the “usual behaviour in a week” question in GPAQ—of course, it could also be that they underestimate walking in the travel diary, but this would be less likely as the diary is an easier recall task. It could also be that people might be walking within larger complexes/buildings or do not consider some kinds of walking as trips. This observation underscores the need for objective PA measures, at least in the survey subsample, alongside these survey instruments. Readily available tools such as pedometers and smartphones would provide basic data for triangulation. In addition, qualitative studies might clarify how people understand these questions.

### 4.3. Study Limitations

Despite filling an important gap in the literature, our study does have limitations, most of which are associated with the data. Firstly, the GPAQ and travel diary are exposed to different levels of recall bias. The GPAQ asks about usual daily and weekly walking/cycling times that are above 10 min, which triggers a different trip recall and averaging process in the responders, compared to the travel diary that focuses on the preceding day and aids trip recall by associating the purpose for leaving home. Secondly, the data collection was conducted around the time of the COVID-19 pandemic, which affected urban mobility in most cities around the world in many ways. The measures implemented to reduce the spread of the COVID-19 pandemic in Accra, such as the stay-at-home orders, travel restrictions, and social distancing, could lead to increased walking and cycling as these became the most convenient/available means for movement, albeit with an overall reduction in walking. While responders would report routine walking/cycling based on their activities before the pandemic in the GPAQ, the dairy reported the preceding day’s walking/cycling, which was more likely affected by COVID-19. Finally, our data findings are only relevant for those aged 15+ years (as constrained by the GPAQ) and may not apply to children and adolescents (<14 years), for whom travel PA is crucial.

## 5. Conclusions

Low PA is prevalent in Accra, Ghana, particularly among females. The travel diary and GPAQ have similar population-level walking estimates when we scale down the GPAQ to expected daily walking. However, there is poor correlation and agreement between individual daily walking durations from both instruments, with the GPAQ reporting higher walking durations. The degree of agreement does not seem to be primarily influenced by large day-to-day fluctuations in travel durations. Our findings also underscore the need to accompany PA questionnaires with objective measures to help calibrate the questionnaires.

## Figures and Tables

**Table 1:** Characteristics of participants surveyed for travel behaviour and physical activity stratified by sex in Accra, Ghana (2020/2021).

	Overall	Female	Male
Number (%)	863 (100)	557 (65)	306 (35)
Mean age (standard deviation)	35 (16)	35 (15)	37 (17)
Age group (%)			
15–24	259 (30)	162 (29)	97 (32)
25–34	209 (24)	146 (26)	63 (21)
35–44	172 (20)	118 (21)	54 (18)
45–54	101 (12)	65 (12)	36 (12)
55–64	68 (8)	36 (7)	32 (11)
65+	54 (6)	30 (5)	24 (8)
≥Secondary school (%)	416 (51)	236 (45)	180 (61)
No occupation (%)	159 (18)	112 (20)	47 (15)
Household has any vehicle (%)	178 (21)	80 (14)	98 (32)
Household has a motorised vehicle (%)	130 (15)	67 (12)	63 (21)

**Table 2:** Physical activity volume by age and gender from GPAQ in the city of Accra.

	Female (Number = 557)			Male (Number = 306)		
	High PA	Medium PA	Low PA	High PA	Medium PA	Low PA
Median MET—hours/week (IQR)	178 (96–258)	23 (14–42)	4 (0–8)	158 (104–302)	26 (16–42)	5 (0–9)
Age group (number and gender row%)						
15–24	29 (18)	50 (31)	83 (51)	37 (38)	26 (27)	34 (35)
25–34	37 (25)	46 (32)	63 (43)	28 (44)	17 (27)	18 (29)
35–44	31 (26)	36 (31)	51 (43)	20 (37)	19 (35)	15 (28)
45–54	11 (17)	19 (29)	35 (54)	13 (36)	10 (28)	13 (36)
55–64	7 (19)	9 (25)	20 (56)	10 (31)	7 (22)	15 (47)
65+	1 (3)	4 (13)	25 (83)	0 (0)	7 (29)	17 (71)
Total (gender row%)	116 (21%)	164 (29%)	277 (50%)	108 (35%)	86 (28%)	112 (37%)

MET: Metabolic equivalent of task; LQ-UQ: lower quartile to upper quartile; IQR: interquartile range.

**Table 3:** Total and domain-specific durations of MVPA and sedentary behaviour by age and sex from GPAQ in Accra, Ghana.

Domain	Age Group (and Total Participants in Age Group)	Participants Reporting a Domain (% of 863)	Mean (LQ-UQ)			Median (IQR) (Only Those with PA)		
			Both Sexes	Female	Male	Both Sexes	Female	Male
All MVPA (minutes/day)	15–24 (259)	230 (30)	108 (13–129)	80 (9–90)	154 (18–176)	32 (116)	26 (81)	47 (158)
	25–34 (209)	187 (24)	149 (13–210)	129 (11–148)	196 (26–304)	43 (197)	36 (137)	86 (278)
	35–44 (172)	151 (20)	159 (13–239)	157 (12–278)	163 (26–209)	64 (226)	51 (266)	86 (183)
	45–54 (101)	86 (12)	123 (11–129)	103 (10–111)	157 (17–209)	34 (117)	26 (101)	55 (192)
	55–64 (68)	59 (8)	139 (10–157)	105 (12–108)	177 (8–212)	29 (147)	26 (95)	36 (204)
	65+ (54)	33 (6)	24 (0–21)	22 (0–14)	26 (0–33)	7 (21)	4 (14)	11 (33)
	Total (863)	746 (86)	127 (11–150)	110 (9–120)	157 (17–208)	34 (139)	30 (111)	54 (191)
Work MVPA (minutes/day)	15–24 (259)	42 (5)	33 (0–0)	27 (0–0)	43 (0–0)	163 (312)	60 (317)	214 (309)
	25–34 (209)	72 (8)	87 (0–69)	80 (0–71)	104 (0–58)	214 (280)	208 (259)	266 (404)
	35–44 (172)	65 (8)	93 (0–132)	98 (0–148)	82 (0–116)	214 (270)	257 (320)	154 (283)
	45–54 (101)	29 (3)	72 (0–15)	60 (0–14)	92 (0–45)	227 (231)	231 (270)	227 (129)
	55–64 (68)	19 (2)	88 (0–64)	48 (0–0)	133 (0–180)	283 (291)	279 (231)	351 (311)
	65+ (54)	3 (0)	2 (0–0)	3 (0–0)	0 (0–0)	9 (32)	9 (32)	NA
	Total (863)	230 (27)	65 (0–14)	60 (0–13)	74 (0–14)	208 (290)	206 (296)	214 (309)
Transport MVPA (minutes/day)	15–24 (259)	221 (26)	65 (10–62)	50 (9–58)	90 (13–120)	30 (75)	26 (46)	43 (111)
	25–34 (209)	171 (20)	51 (6–60)	45 (6–58)	66 (13–69)	30 (56)	27 (50)	40 (64)
	35–44 (172)	139 (16)	60 (6–103)	56 (6–99)	69 (11–103)	39 (104)	30 (107)	60 (90)
	45–54 (101)	77 (9)	44 (4–51)	40 (0–43)	51 (10–64)	26 (64)	25 (56)	29 (62)
	55–64 (68)	57 (7)	47 (8–35)	53 (12–34)	40 (3–42)	26 (39)	22 (26)	26 (34)
	65+ (54)	31 (4)	16 (0–20)	12 (0–12)	21 (0–27)	13 (21)	11 (19)	26 (17)
	Total (863)	696 (81)	54 (6–60)	47 (6–51)	66 (11–77)	30 (65)	26 (56)	36 (80)
*Walking* (minutes/day)	15–24 (259)	220 (25)	61 (10–60)	49 (9–58)	80 (13–114)	29 (71)	26 (46)	30 (111)
	25–34 (209)	171 (20)	50 (6–60)	44 (6–51)	63 (11–69)	30 (56)	27 (47)	40 (66)
	35–44 (172)	137 (16)	59 (6–103)	56 (6–99)	66 (8–100)	39 (105)	30 (107)	60 (91)
	45–54 (101)	76 (9)	42 (3–43)	39 (0–34)	48 (9–54)	26 (53)	25 (56)	27 (45)
	55–64 (68)	57 (7)	43 (7–34)	53 (12–34)	32 (3–31)	26 (39)	22 (26)	26 (39)
	65+ (54)	31 (4)	16 (0–20)	12 (0–12)	21 (0–27)	13 (21)	11 (19)	26 (17)
	Total (863)	692 (80)	51 (6–60)	47 (6–51)	60 (9–64)	29 (64)	26 (52)	30 (73)
*Cycling* (minutes/day)	15–24 (259)	26 (3)	4 (0–0)	0 (0–0)	10 (0–0)	13 (17)	6 (9)	13 (17)
	25–34 (209)	6 (1)	1 (0–0)	0 (0–0)	4 (0–0)	20 (30)	43 (0)	12 (20)
	35–44 (172)	7 (1)	1 (0–0)	0 (0–0)	3 (0–0)	17 (28)	9 (0)	26 (26)
	45–54 (101)	6 (1)	2 (0–0)	1 (0–0)	3 (0–0)	28 (11)	24 (6)	28 (14)
	55–64 (68)	4 (0)	4 (0–0)	0 (0–0)	9 (0–0)	30 (54)	NA	30 (54)
	65+ (54)	0 (0)	0 (0–0)	0 (0–0)	0 (0–0)	NA	NA	NA
	Total (863)	70 (8)	2 (0–0)	0 (0–0)	6 (0–0)	17 (26)	17 (19)	17 (27)
Recreation MVPA (minutes/day)	15–24 (259)	70 (8)	10 (0–4)	3 (0–0)	21 (0–21)	17 (29)	9 (16)	26 (56)
	25–34 (209)	48 (6)	11 (0–0)	4 (0–0)	26 (0–17)	17 (36)	13 (21)	26 (49)
	35–44 (172)	31 (4)	5 (0–0)	2 (0–0)	13 (0–16)	17 (17)	17 (18)	26 (26)
	45–54 (101)	24 (3)	7 (0–0)	3 (0–0)	14 (0–17)	17 (24)	9 (11)	26 (24)
	55–64 (68)	12 (1)	4 (0–0)	4 (0–0)	4 (0–0)	17 (14)	13 (37)	17 (9)
	65+ (54)	11 (1)	6 (0–0)	6 (0–0)	5 (0–9)	9 (17)	95 (91)	9 (13)
	Total (863)	196 (23)	8 (0–0)	4 (0–0)	17 (0–17)	17 (26)	13 (21)	26 (36)
Sedentary behaviour (minutes/day)	15–24 (259)	256 (30)	270 (120–360)	276 (124–360)	261 (120–330)	240 (229)	240 (214)	240 (218)
	25–34 (209)	206 (24)	244 (120–330)	249 (120–360)	232 (120–240)	210 (232)	225 (240)	180 (120)
	35–44 (172)	170 (20)	238 (120–300)	246 (120–352)	222 (120–300)	180 (180)	210 (240)	180 (180)
	45–54 (101)	98 (11)	320 (180–420)	336 (180–480)	291 (180–315)	300 (262)	300 (300)	240 (135)
	55–64 (68)	67 (8)	276 (120–360)	282 (165–368)	268 (120–360)	270 (225)	300 (202)	240 (225)
	65+ (54)	54 (6)	378 (240–480)	381 (218–480)	373 (300–435)	330 (240)	360 (262)	300 (135)
	Total (863)	851 (99)	271 (120–360)	276 (120–360)	261 (150–300)	240 (240)	240 (240)	240 (158)

LQ-UQ: lower quartile to upper quartile; IQR: interquartile range.

**Table 4:** Total and mode–specific transport physical activity by age and gender from the travel diary in Accra, Ghana.

Domain	Age Group (and Total Participants in Age Group)	Participants Reporting a Domain (% of 863)	Mean (LQ-UQ)			Median (IQR) (Only for Travellers)		
			Both Sexes	Female	Male	Both Sexes	Female	Male
Transport PA (minutes/day)	15–24 (259)	160 (19)	34 (0–40)	27 (0–35)	45 (0–50)	35 (40)	30 (32)	40 (61)
	25–34 (209)	127 (15)	38 (0–50)	44 (0–50)	24 (0–42)	40 (40)	40 (50)	36 (38)
	35–44 (172)	97 (11)	36 (0–40)	40 (0–30)	29 (0–49)	30 (40)	28 (44)	40 (35)
	45–54 (101)	51 (6)	20 (0–35)	21 (0–35)	18 (0–35)	35 (40)	38 (40)	35 (30)
	55–64 (68)	34 (4)	24 (0–20)	20 (0–20)	29 (0–16)	20 (33)	20 (26)	30 (40)
	65+ (54)	20 (2)	17 (0–14)	6 (0–4)	31 (0–52)	32 (50)	20 (14)	60 (55)
	Total (863)	489 (57)	32 (0–40)	32 (0–39)	32 (0–44)	35 (40)	30 (44)	40 (40)
Walking (minutes/day)	15–24 (259)	160 (19)	34 (0–40)	27 (0–35)	45 (0–50)	35 (40)	30 (30)	40 (61)
	25–34 (209)	125 (14)	37 (0–47)	44 (0–50)	22 (0–38)	40 (40)	40 (50)	36 (39)
	35–44 (172)	94 (11)	36 (0–36)	40 (0–30)	27 (0–44)	30 (43)	28 (44)	40 (35)
	45–54 (101)	51 (6)	20 (0–35)	21 (0–35)	17 (0–35)	35 (40)	38 (40)	35 (24)
	55–64 (68)	32 (4)	18 (0–20)	20 (0–20)	15 (0–10)	20 (30)	20 (26)	20 (25)
	65+ (54)	20 (2)	17 (0–14)	6 (0–4)	31 (0–52)	32 (50)	20 (14)	60 (55)
	Total (863)	482 (56)	31 (0–40)	32 (0–39)	30 (0–40)	35 (40)	30 (44)	38 (41)
Cycling (minutes/day)	15–24 (259)	1 (0)	0 (0–0)	0 (0–0)	0 (0–0)	20 (0)	20 (0)	<NA>
	25–34 (209)	2 (0)	0 (0–0)	0 (0–0)	1 (0–0)	42 (18)	<NA>	42 (18)
	35–44 (172)	2 (0)	1 (0–0)	0 (0–0)	2 (0–0)	30 (15)	<NA>	30 (15)
	45–54 (101)	1 (0)	0 (0–0)	0 (0–0)	0 (0–0)	15 (0)	<NA>	15 (0)
	55–64 (68)	2 (0)	6 (0–0)	0 (0–0)	14 (0–0)	220 (170)	<NA>	220 (170)
	65+ (54)	0 (0)	0 (0–0)	0 (0–0)	0 (0–0)	0 (0)	<NA>	<NA>
	Total (863)	8 (1)	1 (0–0)	0 (0–0)	2 (0–0)	30 (30)	20 (0)	40 (29)

LQ-UQ: lower quartile to upper quartile; IQR: interquartile range.

**Table 5:** Walking in the travel diary compared to walking in the GPAQ.

		GPAQ			
		No Walk (Number = 169)	Walked for ≥1 Day (Number = 694)	Walked for ≥5 Days (Number = 414)	Walked for 7 Days (Number = 181)
Travel Diary	No walk (number = 3 81)	126	255	124	54
	Walked (number = 482)	43	439	290	127

**Table 6:** Comparison of daily walking duration (minutes) between GPAQ and travel diary.

Participant Group	GPAQ Mean Daily Walking Duration (Standard Deviation)	Diary Mean Daily Walking Duration (Standard Deviation)	Bias (95% Limits of Agreement)	Percentage of GPAQ > Diary	Rho (95% Confidence Limits)
All participants	51 (82)	31 (65)	20 (–151 to 192)	54	0.31 (0.25 to 0.37)
Walk ≥ 1 day GPAQ	64 (87)	36 (69)	28 (–157 to 213)	68	0.29 (0.22 to 0.35)
Walk ≥ 5 days GPAQ	87 (101)	45 (79)	45 (–175 to 265)	70	0.24 (0.15 to 0.33)
Walk = 7 days GPAQ	98 (102)	40 (77)	57 (–179 to 294)	73	0.11 (–0.04 to 0.27)

## Data Availability

The datasets used and analysed during the current study are available on reasonable request from the author listed last in this article.
